# Biological Evaluation of Dinuclear Platinum(II) Complexes with Aromatic *N*-Heterocycles as Bridging Ligands

**DOI:** 10.3390/ijms25158525

**Published:** 2024-08-05

**Authors:** Desimir Luković, Andjela A. Franich, Marija D. Živković, Snežana Rajković, Bojan Stojanović, Nevena Gajović, Milena Jurišević, Slađana Pavlović, Bojana Simović Marković, Marina Jovanović, Bojana S. Stojanović, Radiša Pavlović, Ivan Jovanović

**Affiliations:** 1 Center for Molecular Medicine and Stem Cell Research, Faculty of Medical Sciences, University of Kragujevac, S. Markovića 69, 34000 Kragujevac, Serbia; desimir.lukovic79@gmail.com (D.L.); gajovicnevena@yahoo.com (N.G.); milena.jurisevic13@gmail.com (M.J.); sladjadile@gmail.com (S.P.); bojana.simovic@gmail.com (B.S.M.); marina_jovanovic@rocketmail.com (M.J.); bojana.stojanovic04@gmail.com (B.S.S.); ivanjovanovic77@gmail.com (I.J.); 2 Department of Chemistry, Faculty of Science, University of Kragujevac, R. Domanovića 12, 34000 Kragujevac, Serbia; andjela.frenich@pmf.kg.ac.rs (A.A.F.); snezana.rajkovic@pmf.kg.ac.rs (S.R.); 3 Department of Pharmacy, Faculty of Medical Sciences, University of Kragujevac, S. Markovića 69, 34000 Kragujevac, Serbia; 4 Department of Surgery, Faculty of Medical Sciences, University of Kragujevac, S. Markovića 69, 34000 Kragujevac, Serbia; 5 Department of Clinical Pharmacy, Faculty of Medical Sciences, University of Kragujevac, S. Markovića 69, 34000 Kragujevac, Serbia; r.pavlovic2407@gmail.com; 6 Department of Pathophysiology, Faculty of Medical Sciences, University of Kragujevac, S. Markovića 69, 34000 Kragujevac, Serbia

**Keywords:** dinuclear platinum(II) complexes, antitumor effect, apoptosis, cell proliferation, DNA interaction, biological evaluation, cancer therapy

## Abstract

The history of effective anti-cancer medications begins with the discovery of cisplatin’s anti-cancer properties. Second-generation analogue, carboplatin, with a similar range of effectiveness, made progress in improving these drugs with fewer side effects and better solubility. Renewed interest in platinum-based drugs has been increasing in the past several years. These developments highlight a revitalized enthusiasm and ongoing exploration in platinum chemotherapy based on the series of dinuclear platinum(II) complexes, [{Pt(L)Cl}_2_(*μ*-bridging ligand)]^2+^, which have been synthesized and evaluated for their biological activities. These complexes are designed to target various cancerous conditions, exhibiting promising antitumor, antiproliferative, and apoptosis-inducing activities. The current work aims to shed light on the potential of these complexes as next-generation platinum-based therapies, highlighting their enhanced efficacy and reduced side effects, which could revolutionize the approach to chemotherapy.

## 1. Introduction

The medicinal application of metals can be traced back almost 5000 years, but the development of modern medicinal inorganic chemistry was stimulated by the discovery of cisplatin in 1969 [1]. Cisplatin (*cis*-diamminedichloroplatinum(II), platinol, Table 1) is the most well-known conventional platinum anticancer complex which effectively treats testicular, ovarian, head, neck and small/cell lung cancer [2]. Cisplatin binds the covalent to DNA, after undergoing a ligand substitution where a chloride ion is replaced by a water molecule, blocking transcription and replication, which initiates the apoptosis process in cells [3,4]. This reaction occurs more readily in the cytoplasm than in the bloodstream, due to lower chloride concentrations [5]. Once aquated, cisplatin interacts with DNA, preferentially binding to guanine bases and forming intrastrand cross-links, which can disrupt DNA structure and lead to cell death if not repaired. DNA repair mechanisms, particularly nucleotide excision repair, can influence the effectiveness of cisplatin. Proteins that bind to distorted DNA, like the high-mobility group box proteins, might shield platinum adducts from repair, affecting treatment outcomes. In addition, platinum drugs can interact with blood proteins and cellular detoxification systems, contributing to drug resistance [6,7,8].

Nevertheless, the application of cisplatin is limited, due to the characteristic side effects such as emetogenicity, ototoxicity, nephrotoxicity and neurotoxicity, as well as increasing resistance in tumor cells [9,10,11]. The clinical limitations of cisplatin have been the motivation for the creation of cisplatin analogues.

Carboplatin ([Pt(CBDCA-*O*,*O*’)(NH_3_)_2_], paraplatin, Table 1) has been used in oncotherapy since 1989 [12]. It has fewer toxic side effects than cisplatin and is more easily used in combination therapy, due to the slower rate of conversion of carboplatin to reactive species. Because of its low reactivity, carboplatin is better tolerated by patients and can be administrated at higher doses than cisplatin. Carboplatin is mostly used for ovarian cancer, lung cancer, head and neck cancer, brain cancer, and neuroblastoma treatment [13]. Studies on the interaction of carboplatin with DNA indicate the same mode of action as cisplatin (covalent binding to DNA), suggesting identical products formed by cisplatin and carboplatin interaction with DNA [14]. Cross-links formed after guanine base binding, can occur between guanines on the same DNA strand (intrastrand) or across different strands (interstrand). The most common intrastrand cross-link is 1,2-d(GpG), making up 65% of the lesions, followed by 1,2-(ApG) and 1,3-d(GpTpG) in lower percentages, with some GG interstrand cross-links also present. Cisplatin and carboplatin form similar cross-links, though in different proportions. These cross-links significantly alter the DNA structure, causing bending and unwinding of the double helix, as shown by atomic resolution studies of various platinum drug adducts [14,15]. Unfortunately, resistance of tumor cells to carboplatin was also observed in the same way as with cisplatin [16]. 

Oxaliplatin (*trans*-*R*,*R*-cyclohexane-(1,2-diamine), eloxatin, Table 1), as a drug, was used for the first time in France in 1996, and licensed for Europe in 1999 and for the US in 2002, 23 years after being patented [13]. Oxaliplatin was approved for the treatment of colorectal cancer [17]. It was the first platinum(II)-based anticancer drug to overcome tumor cell resistance. However, a major oxaliplatin side effect of oxaliplatin is neurotoxicity, which limits the dose of the drug [18]. DNA-binding products of oxaliplatin are similar, but not identical, to those formed by cisplatin and carboplatin. The main advantage of oxaliplatin efficiency is that it binds to DNA by covering major groove and prevents the binding of proteins for the reparation of the DNA chain [19,20,21].

In 1983, the Japanese company Shionogi Pharmaceutical Company, located in Osaka, Japan, designed nedaplatin (*cis*-diammine-glycolatoplatinum(II), aqupla, Table 1) [22]. The use of nedaplatin in medicine began in 1995 in Japan. Nedaplatin is used to treat head and neck, esophageal, and lung tumors [23]. As the glycolato chelate ligand increases the solubility of nedaplatin in water, this drug is administered intravenously in a higher dose than cisplatin [24]. Nedaplatin, a cisplatin analog (Table 1), shows cross-resistance with cisplatin. It forms nucleoside–platinum complexes similarly to cisplatin and, after cellular uptake and hydrolysis, binds to DNA to inhibit its replication. The primary dose-limiting side effect of nedaplatin is myelosuppression, including issues like leucopenia, anemia, and, mainly, thrombocytopenia [25]. Histopathological studies in rats have shown that nedaplatin can cause nephrotoxicity, characterized by apoptosis or necrosis in both proximal and distal renal tubules, as well as the collecting duct, with potential regeneration and cystic dilatation [26]. These findings suggest that while nedaplatin can cause kidney damage, particularly in patients with pre-existing renal issues, strategies can be developed to use it safely in clinical settings [24].

Heptaplatin (*cis*-malonate[(4*R*,5*R*)-4,5-bis(aminomethyl)-2-isopropyl-1,3-dioxolane]platinum(II), SunPla, Table 1) entered the market in 1999 through the Japanese pharmaceutical company Yakult Honsha Co., Ltd. (Tokyo, Japan), and is used for the treatment of colorectal tumors in the Republic of Korea [27]. Clinical investigation showed that the combination of heptaplatin with 5-fluorouracil has the same effect as the combination of cisplatin and 5-fluorouracil, with fewer hematological side effects [21,23]. Heptaplatin was chosen for clinical trials due to its comparable or superior cytotoxicity to cisplatin in various cell lines, high stability, minimal toxicity, and effectiveness against cisplatin-resistant cancer cells. In clinical trials, heptaplatin showed improved response rates when combined with 5-fluorouracil and leucovorin compared to its use alone, and lower nephrotoxicity than cisplatin. Overall, response and survival rates with heptaplatin are similar to those with cisplatin, but with reduced severity of certain side effects like neutropenia and proteinuria [23].

However, heptaplatin showed side effects such as myelosuppression, thrombocytopenia, mucositis and alopecia [23]. Heptaplatin binds to DNA by forming covalent adducts with DNA bases, leading to crosslink and inhibition of DNA replication and transcription [27]. 

Lobaplatin ([*1R*,*2R*-2-(aminomethyl)cyclobutyl]methanamine-2-hydroxypropanoic acid platinum(II), Table 1) is approved in China for the treatment of breast tumor metastasis, chronic myelogenous leukemia and lung cancer [12]. Newly formed DNA-drug adducts such as GG and AG intra-strand crosslinks affect the expression of the *c-Myc* gene, which is responsible for apoptosis [28]. Lobaplatin has been efficient against tumor cells that are resistant to cisplatin [29]. The dose-limiting toxicity of lobaplatin is reflected in thrombocytopenia [23].

Regardless of unwanted effects of chemotherapeutic agents, it is evident that platinum-based compounds are an essential component of contemporary cancer-intervention strategies and the demand for platinum antitumor agents constantly grows. Listed clinically used drugs are neutral square-planar complexes of platinum(II), which contain two outgoing ligands in the *cis*-position (classical platinum complexes) [30,31,32]. In order to find a platinum complex with more efficient antitumor activity, non-classical platinum complexes that cannot be structurally connected to cisplatin were synthesized and tested [33]. Platinum(II) complexes that are structurally different from cisplatin and its analogs provide numerous opportunities for finding antitumor agents whose mechanism of action will be different, compared to cisplatin [34,35,36]. 

Beside mononuclear platinum(II) and platinum(IV) complexes, polynuclear platinum(II) complexes show antitumor activity against certain types of cancerous diseases [37,38]. Polynuclear complexes usually contain from two to four platinum(II) ions, which are interconnected by various bridging diamine ligands. These bridging diamine ligands are often flexible molecules with a linear structure [39], while in some cases platinum(II) ions are interconnected with less-flexible bridging ligands, such as aromatic heterocyclic compounds, which contain two or more nitrogen atoms in the ring [40,41]. Polynuclear platinum(II) complexes can bind to nucleic bases in the DNA strand, forming platinum(II) DNA products that are structurally different from those formed by cisplatin and similar complexes, leading to minimal distortion of the DNA helix [42]. Thanks to the presence of two platinum(II) centers, dinuclear platinum(II) complexes cause two different types of binding, intermolecular binding between DNA strands and intramolecular binding within the DNA strand [43]. 

The trinuclear complex, Triplatin (or BBR3464), showed better antitumor activity and lower tumor-cell resistance compared to cisplatin, regardless of the fact that a different way of binding to DNA has been proven [44,45]. Complex Triplatin does not have the potential for covalent binding, intercalation or groove binding. Instead, the positively charged BBR3464 complex (+4) binds to the phosphate backbone, through the electrostatic interaction [46]. These polynuclear platinum(II) complexes with flexible aliphatic ligands demonstrate greater effectiveness than cisplatin in treating LNZ308 and LN443 glioma cells, as well as HCT-116, DLD1, SW480, and HT29 colon cancer cells in both culture and animal models [47]. Although patients with neuroblastoma and ovarian tumor did not show resistance to the treatment with trinuclear BBR3464 complex, they had gastrointestinal and hematological side effects that limit the dose of the used complex. [48,49]. Additionally, the pyrazine-bridged dinuclear platinum(II) complex shows equal or superior cytotoxicity compared to cisplatin in WIDR colon and IGROV ovarian cancer cell lines, and exhibits significant effectiveness against the cisplatin-resistant L1210 murine leukemia cell line [47].

Therefore, the synthesis and investigation of polynuclear platinum(II) complexes are important steps in the field of antitumor-agent improvement. This review article focuses on recent advances in dinuclear platinum(II) complexes as future anticancer agents. The dinuclear platinum(II) complexes exhibit a potent antitumor effect, selectively targeting cancer cells over normal cells. Furthermore, their ability to induce apoptosis in cancer cells, a critical mechanism for cancer therapy, underscores their potential as effective anticancer agents. Recent studies have demonstrated that these complexes can significantly reduce tumor-cell viability and proliferation, indicating their promise in enhancing current chemotherapy strategies. 

## 2. Synthesis of Dinuclear Platinum(II) Complexes, [{Pt(L)Cl}_2_(*μ*-Bridging Ligand)]^2+^

**Figure 1:**
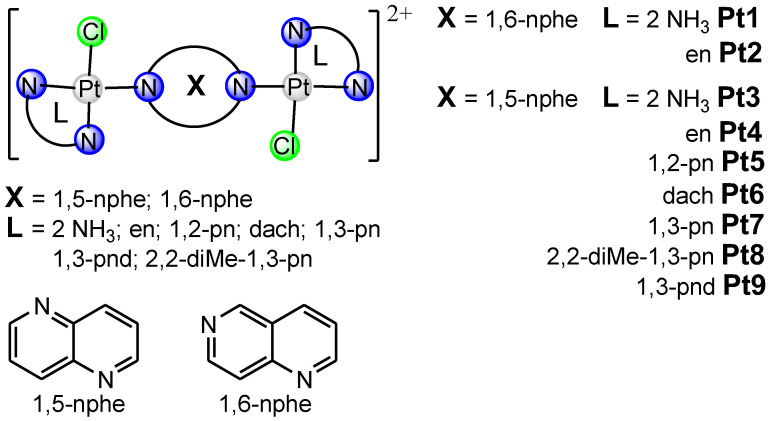
The structural formula of dinuclear platinum(II) complexes containing bridging ligand with two condensed aromatic rings with two nitrogen atoms in two different rings (Pt1–Pt9).

**Figure 2:**
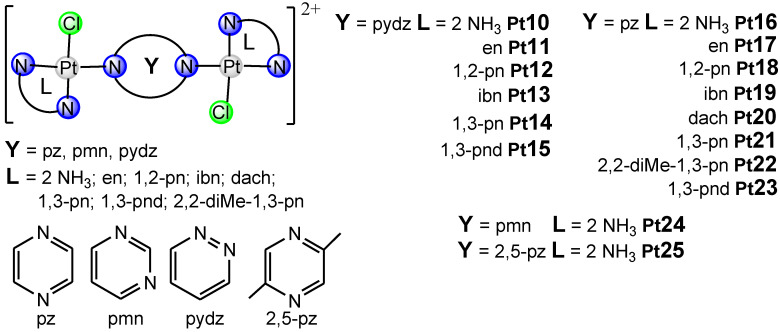
The structural formula of dinuclear platinum(II) complexes containing bridging ligand with one aromatic ring with two nitrogen atoms (Pt10–Pt25).

**Figure 3:**
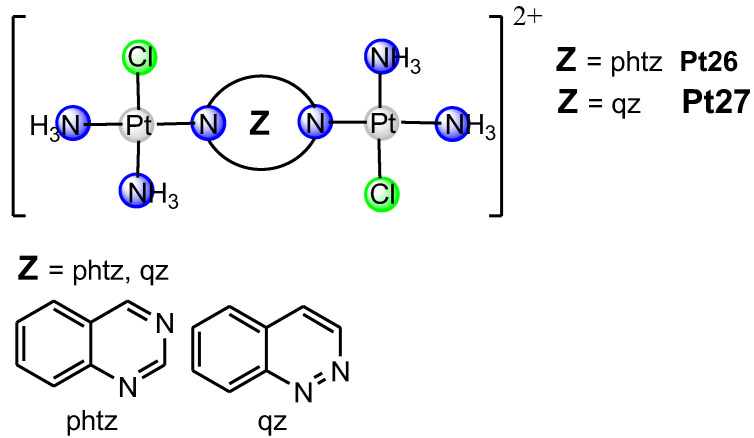
The structural formula of dinuclear platinum(II) complexes containing bridging ligand with two condensed aromatic rings with two nitrogen atoms in one ring (Pt26–Pt27).

**Figure 4:**
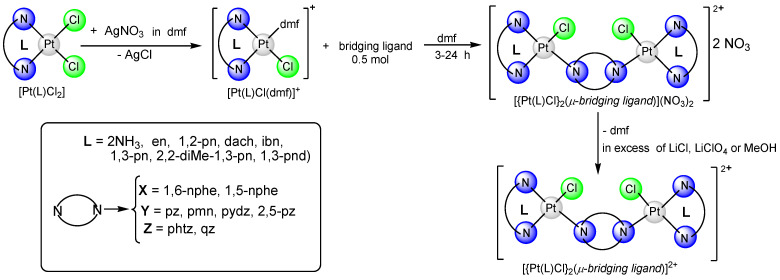
Schematic presentation of reactions for preparation of {[Pt(L)Cl]_2_(*μ*-bridging ligand)}^2+^ complex.

In this review article, Figure 1, Figure 2 and Figure 3 depict various dinuclear platinum(II) complexes with the general formula [{Pt(L)Cl}_2_(*μ*-bridging ligand)]^2+^. Figure 4 illustrates the synthesis process for diazine-bridged dinuclear platinum(II) complexes. Mononuclear [Pt(L)Cl_2_] complexes, where L represents either two monodentate ammine ligands (NH_3_) or a single bidentate diamine ligand (such as ethylenediamine, en; (±)-1,2-propylenediamine, 1,2-pn; isobutylenediamine, ibn; *trans*-(±)-1,2-diaminocyclohexane, dach; 1,3-propylenediamine, 1,3-pn; 2,2-dimethyl-1,3-propylenediamine, 2,2-diMe-1,3-pn and 1,3-pentanediamine 1,3-pnd), were synthesized following established procedures [50,51,52,53,54]. The synthesis reaction starts with the formation of the mononuclear [Pt(L)I_2_] complex by addition of the four equivalents of potassium iodide in a aqueous solution of K_2_[PtCl_4_], followed by heating the mixture at 40 °C for 5 min. After the addition of an equimolar amount of diamine ligand (L), stirring continued for 30 min at the same temperature. The resulting [Pt(L)I_2_] complexes were converted into aqua derivatives by adding 1.98 equivalents of AgNO_3_ and stirring overnight at room temperature in the dark [54]. After filtration to remove AgCl, an excess of potassium chloride was added to form mononuclear [Pt(L)Cl_2_] complexes.

These mononuclear complexes then reacted with 0.98 equivalents of AgNO_3_ to replace one chloride ion with a dmf. To obtain the dinuclear {[Pt(L)Cl]_2_(*μ*-bridging ligand)}^2+^ complexes, the resulting [Pt(L)Cl)dmf)]^+^ complex, obtained after AgCl removal, reacted with an equivalent amount of the corresponding bridging ligand (X, Y, or Z, as shown in Figure 1, Figure 2 and Figure 3) and were stirred at room temperature in the dark for 3–24 h.

The bridging ligands X include two condensed aromatic rings with nitrogen atoms in different rings (e.g., 1,5-naphthyridine, 1,5-nphe [55]; 1,6-naphthyridine, 1,6-nphe [56] for complexes Pt1–Pt9, Figure 1), Y contain a single aromatic ring with two nitrogen atoms (e.g., pyrazine (1,4-diazine), pz [57]; 2,5-dimethylpirazine (2,5-dimethyl-1,4-diazine), 2,5-pz [58], pyrimidine (1,3-diazine), pmn [58]; pyridazine (1,2-diazine), pydz [59] for complexes Pt10–Pt25, Figure 2), and Z have two condensed aromatic rings with nitrogen atoms in one (e.g., phthalazine (2,3-benzodiazine), phtz; quinazoline (1,3-benzodiazine), qz [58] for complexes Pt26 and Pt27, Figure 3). 

The dinuclear platinum(II) complexes were crystallized as chloride, nitrate or perchlorate salts from aqueous solutions with excess of LiCl, LiClO_4_ or by evaporating methanol solvent for nitrate salts (Pt10, Pt16 and Pt24). Detailed procedures for the preparation of all dinuclear platinum(II) complexes have been previously described [55,56,57,58,59,60].

## 3. Dinuclear Platinum(II) Complexes Have a Potent Antitumor Effect In Vitro

This article presents the pivotal anticancer activity mechanisms in vitro of the dinuclear platinum(II) complexes with 1,6-naphthyridine-bridging ligand described in the literature to date. Derivatives of 1,6-naphthyridine have demonstrated noteworthy biological activity, attracting the interest of pharmacists because of their lower toxicity. Several of these compounds are utilized in drugs for preventing and treating infections caused by various bacteria [61]. Additionally, derivatives like 1,4-dihydro-4-oxo-1,6-naphthyridine and 8-methylbenzo[b]naphtho [1,6]-naphthyridine have shown antibacterial properties [62]. Numerous 1,6-naphthyridine derivatives are being explored as potential anticancer agents, and some have shown promise for antimalarial and antidiabetic applications [63]. For instance, 5-substituted 8-hydroxy-1,6-naphthyridine-7-carboxamides are effective as HIV integrase inhibitors for treating HIV infection [61]. The presence of naphthyridine in these compounds and its impact on their antibacterial or antitumor activities has led to the synthesis and investigation of various transitional metal complexes.

Studies on cell lines indicate that metal complexes, especially the dinuclear platinum(II) complexes, are selective for cancer cells [55,56,57,58,59,64,65]. Much effort has been put into the development of new platinum-based anticancer complexes, but none have reached worldwide clinical application so far. Since it was revealed that dinuclear platinum(II) complexes with 1,6-naphthyridine-bridging ligand play an important role in the biology of cancers, we have summarized current knowledge about the cytotoxic abilities of these complexes.

Recently, we demonstrated [56] high cytotoxic activity of dinuclear platinum(II) complexes with 1,6-naphthyridine-bridging ligand against mouse breast (4T1) and colon (CT26) cancer cell lines, and human breast (MDA-MB-468), colon (HCT-116), and lung (A549) cancer cell lines. Dinuclear platinum(II) complexes with 1,6-naphthyridine as the bridging ligand reduced the viability of all tested cancer cell lines, although the established cytotoxic effects were less compared to cisplatin as the gold standard [56]. Among all tested newly synthesized dinuclear platinum(II) complexes, it appears that [{PtCl(NH_3_)_2_}_2_(*μ*-1,6-nphe)]^2+^ (Pt1, Figure 1) may cause fewer side effects compared to cisplatin. In contrast, [{Pt(en)Cl}_2_(*μ*-1,6-nphe)]^2+^ (Pt2, Figure 1) complex had non-significant cytotoxic activity against tested cancer cell lines with IC_50_ values of 281.03 μM and higher [56]. Furthermore, based on the calculation of selectivity index (SI) values, the SI values for Pt1 and Pt2 complexes were at least nine times higher than those calculated for cisplatin, implying that Pt1 and Pt2 complexes may have fewer side effects. Despite the fact that different isomeric forms of naphthyridines showed broad biological abilities such as anti-inflammatory, antiviral, antimicrobial and anticancer effect, the main limitation of these studies is the lack of effects on immune cells [64]. Additionally, complexes with iridium(III) and rhodium(III) with 1,8-naphthyridine have cytotoxic activity against colon and breast cancer cell lines [65]. In line with these findings, Konovalov and coworkers [56] recently demonstrated that ligands in dinuclear complexes might play a key role in their cytotoxicity. Newly synthesized Pt1 complex contains two ammonia ligands, whereas Pt2 complex contains ethylenediamine, which are responsible for different cytotoxic effects [56]. Previously, Konovalov et al. [55] investigated seven new 1,5-naphthyridine-bridged (1,5-nphe) dinuclear platinum(II) complexes, and the results confirmed their antitumor role (Pt3–Pt9, Figure 1). Almost all tested complexes had no cytotoxic effects on murine mammary carcinoma cell lines (4T1) and very low cytotoxicity towards murine lung cancer cells (LLC1) [55]. In contrast to the cytotoxic effects on LLC1 and 4T1 cancer cells, complexes with two ammines (Pt3) or one bidentate coordinated diamine (ethylenediamine, Pt4) had significant cytotoxic activity towards CT26 murine colon carcinoma cells [55]. These findings indicated that the dinuclear platinum(II) complexes containing an aromatic 1,5-naphthyridine bridging ligand could be good candidates for therapeutic purposes for colon cancer. In line with these results, dinuclear [{Pt(L)Cl}_2_(*μ*-pydz)]Cl_2_ (Pt10–Pt15, Figure 2) complexes showed high cytotoxic effects in a dosage- and time-dependent manner, correlating the concentration of the tested complexes with the cell viability of tumor cells after 48 h and 72 h against mouse cell lines (4T1, LLC1, and B16F10) and human cell lines (MDA-MB 468, A549, and A375) [59]. The cytotoxic effect of dinuclear Pt10–Pt15 complexes was dose dependent: a concentration decrement in all tested compounds was followed by marked increment in tumor cell viability. The obtained data from this study [59] also revealed that, following 48 h of exposure, Pt13 showed a dose-dependent cytotoxic effect against mouse breast cancer cells (4T1) (IC50 = 146.48 ± 75 μM), while other tested complexes were cytotoxic for these cells only at the highest concentrations (250 and 500 μM). A similar pattern of newly synthesized complexes’ cytotoxicity towards all tested cell lines was determined following 72 h of exposure. Moreover, after 72 h of exposure, the selectivity indices of cisplatin for all tested tumor cell lines were less than 2, indicating general toxicity (5). In another study, Vasic and coworkers [57] showed the cytotoxicity of cationic platinum(II) complexes against the murine colon carcinoma (CT26) cell line. An increase in the cytotoxicity of all tested complexes was observed 72 h after treatment. The highest cytotoxicity against CT26 was exhibited by Pt11 and Pt16–Pt23 (Figure 2), while complex Pt16 managed to kill about 50% of tumor cells at the lowest tested concentration (8.82 μM), which is suitable for in vivo application. Most importantly, this complex showed significantly higher cytotoxicity than oxaliplatin at some tested concentrations [57]. In addition, the cytotoxic effects of azine-bridged complexes (Pt10, Pt16, Pt24–Pt27, (Figure 2 and Figure 3) were also well documented in several human tumor cell lines such as MCF and EVSA-T (breast cancer), WIDR (colon cancer), IGROV (ovarian cancer), M19 (melanoma), A498 (renal cancer), and H226 (non-small-cell lung cancer) [58]. Among the three tested dinuclear compounds (Pt25–Pt27), antitumor effect was lower than for cisplatin [58].

Due to intensive research work, the dinuclear platinum(II) complexes with 1,6-naphthyridine-bridging ligand compounds (Pt1, Pt2), as shown in the present article [55,56,57,58,64,65], demonstrate great potential for application, and may soon be used as anticancer drugs.

## 4. Dinuclear Platinum(II) Complexes Induce Apoptosis of Target Cells

The analysis and elucidation of the molecular mechanisms of cell death has greatly contributed to the insight into the pathogenesis of malignancy, as well as the sensitivity of normal and malignant cells to different types of therapy [66]. Such research enabled the identification of potential targets for new therapeutic procedures. Changes in sensitivity to apoptosis not only contribute to uncontrolled proliferation and the development of malignancy, but can also increase resistance to conventional anti-cancer therapies [67]. Mitochondria-dependent apoptosis is one of the key pathways for the induction of apoptosis [68]. Inhibition or evading of this pathway is an effective way for cancer cells to overcome apoptosis, thus indirectly ensuring mutation accumulation and subsequent uncontrolled cell division [69].

Understanding how this breakdown occurs in cancer cells is still a subject of intense research [70,71]. Numerous tests conducted in our laboratory on various dinuclear platinum(II) complexes have shown their strong potential to induce apoptosis in both murine and human tumor cells. There are several methods available for detecting apoptosis in cancer cells. One of the preferred analyses is the annexin V-FITC-propidium iodide flow cytometric analysis, which involves staining with annexin V that has a high affinity for phosphatidylserine on the outer surface of apoptotic cell membranes, and propidium iodide that can bind to DNA and enter necrotic or late-apoptotic cells [72]. In our previous studies, we analyzed different stages of apoptosis in CT26 mouse colon carcinoma cells treated with platinum(II) complexes and oxaliplatin, using flow cytometry after double staining with annexin V-FITC and propidium iodide 24 h after treatment with all complexes. We have demonstrated that treatment of mouse colon carcinoma CT26 cells with platinum(II) complexes (Pt11, Pt16–Pt23) increases the level of both late and early apoptosis. The highest percentage of apoptotic CT26 cells, particularly early-apoptotic CT26 cells, was observed following the treatment with complexes Pt17, Pt18, and Pt19 [57].

Also, treatment of CT26 tumor cells with Pt3 and Pt4 complexes (Pt3 containing two ammines and Pt4 bidentate coordinated ethylenediamine, while 1,5-nphe is a bridging ligand) increased the sensitivity of tumor cells to apoptosis, both in the early and late stages [55]. In our subsequent research using the same approach, we examined the potency of the Pt13 complex in causing apoptotic cell death in mouse (4T1) and human (MDA-MB 468) breast cancer cells. Exposure to the complex at a concentration of 30 μM induced apoptosis in 4T1 cells, resulting in a significantly higher percentage of early-apoptotic 4T1 cells. In MDA-MB 468 human breast cancer cells, the complex affected cells in both the early and late stages of apoptosis [59].

Cellular stress caused by various stimuli can trigger apoptotic cell death through two signaling pathways: the extrinsic and intrinsic (or mitochondrial) pathways. Once the internal apoptotic pathway is activated, cytochrome c is released from the mitochondria forming the apoptosome and leading to the cleavage of initiator caspase 9 and subsequent activation of effector caspase 3 [73]. The impact of Pt13 on the induction of apoptosis in 4T1 cells was further confirmed by the increased expression of effector caspase 3 mRNA following Pt13 complex treatment. Similarly, significantly increased expression of caspase 9 mRNA in 4T1 cells after exposure to the Pt13 complex indicates that the death of mouse breast cancer cells is mediated by the activation of the intrinsic apoptotic pathway [59].

The Bcl-2 family plays a central role in the apoptotic pathway [74]. The Bcl-2 family of proteins consists of pro- and anti-apoptotic members, and the balance between them maintains the equilibrium between newly formed cells and old cells that die. The balance between pro-survival and pro-apoptotic Bcl-2 family members determines cell survival [75]. Tumor cells can acquire resistance to apoptosis by the expression of anti-apoptotic proteins such as Bcl-2 or by the downregulation or mutation of pro-apoptotic proteins such as BAX [76]. One of the roles of Bcl-2 in the mitochondrial pathway is to regulate the intracellular redox status, favoring a pro-oxidant milieu that ensures survival. In such an environment, the reduced form of cytochrome c is inhibited in its activity to initiate caspase activation [77]. Bcl-2 also can inhibit the activity of caspase-9, 3, 6, and 7, thereby preventing apoptosis, leading to prolonged tumor cell survival and malignant cell transformation [73]. Our recent studies showed that the dinuclear platinum(II) complexes, specifically the Pt1 complex, induce apoptosis in 4T1 and A549 cells by increasing the expression of pro-apoptotic caspase-3 and downregulating the expression of anti-apoptotic Bcl-2 after 24 h pretreatment with the Pt1 complex. This finding presents a significant break-through in understanding how the Pt1 complex triggers apoptosis in cancer cells [56].

The mechanism of apoptosis caused by dinuclear platinum complexes has been the subject of numerous studies. A new class of platinum anticancer compounds, the azine-bridged dinuclear platinum(II) complexes show a cytotoxic effect different from cisplatin. They induce apoptosis in murine leukemia cells and form DNA adducts that are different from those of cisplatin and thus circumvent, to some extent, the cross-resistance with cisplatin [58]. Similarly, TriplatinNC and TriplatinNC-A also show micromolar toxicity against cisplatin-sensitive and cisplatin-resistant ovarian cancer cells. Contrary to TriplatinNC, which induces apoptosis in a manner similar to cisplatin and BBR3464, TriplatinNC-A induces cell death in a manner that is independent of p53- or BAX-status. These agents overcome cisplatin resistance because they accumulate in the cells, probably due to their cationic nature and their unique mode of binding to DNA [15,78]. The aggregates of the platinum(II) complex BDIQQ) [Pt(BDI^QQ^)]Cl in aqueous buffer disperse in the presence of DNA and form single molecules that are capable of unwinding DNA. [Pt(BDI^QQ^)]Cl has a dual mode of action. It attacks DNA in cells, increasing p53 and BAX levels and inducing mitochondria-mediated apoptosis, and it accumulates in mitochondria, causing direct damage to the mitochondria. [Pt(BDIQQ)]Cl selectively damages ovarian cancer cells, while it has no effect on normal fibroblast cells and shows no cross-resistance with cisplatin [15,79]. A mitochondria-mediated apoptotic cell death can also be triggered by replacing a dichloroacetate with a vitamin E analog, α-tocopheryl succinate (a-TOS), which inhibits the anti-apoptotic proteins Bcl-2 and Bcl-xL. Due to high lipophilicity and susceptibility to entrapment inside the cell membrane, platinum(IV) complexes comprising cisplatin attached to two a-TOS ligands was shown to be non-toxic, while with one a-TOS ligand Platinum(IV) complexes inhibited Bcl-xL-Bax protein–protein interactions and thus induced DNA damage and mitochondrial membrane depolarization, and were 25 times more cytotoxic compared to cisplatin [15,80].

## 5. Dinuclear Platinum(II) Complexes Affect Cell Proliferation

The major characteristic responsible for the development of cancer is uncontrolled division of tumor cells. In comparison to healthy cells, which respond according to different stimulative and inhibiting signals, thus controlling the normal cell cycle and the level of proliferation, tumor cells grow and proliferate in an uncontrolled manner, eventually spreading into different tissues and organs [81]. One of the crucial molecules that plays an important role as a marker for cancer histopathology is Ki67. Ki67 is a molecule predominantly placed in fast-dividing cells and is often used for cancer prognosis [82]. A recent study suggested that Ki67 is not only involved in the process of proliferation, but also in the complex process of tumor initiation, growth, metastasis, and drug resistance [83]. Some trials have successfully led to the rapid registration of new anticancer drugs with a significant impact on tumor cell proliferation. As dinuclear platinum(II) complexes with 1,6-naphthyridine-bridging ligand (Pt1, Pt2) show cytotoxic effect against different types of mice and human tumor cell lines, their effect on proliferation was also analyzed. Konovalov et al. showed that dinuclear platinum(II) complexes with 1,6-naphthyridine-bridging ligand significantly decreased the percentage of Ki67^+^ 4T1 mouse breast cancer cells, as well as Ki67^+^ A549 human lung cells, in comparison to the untreated group [56]. A similar experiment was performed in another study on the murine colorectal carcinoma cell line (CT26) [57]. The percentage of Ki67+ CT26 cells was significantly lower after treatment with seven out of nine tested dinuclear platinum (II) complexes, with complexes Pt20 and Pt21 showing the strongest effect compared to cells treated with oxaliplatin [57]. As Ki-67 is nowadays used as an important predictive and prognostic marker, and higher expression correlates with poor survival, treatment modalities with dinuclear platinum (II) complexes against Ki-67 can offer promising results.

The advantage of tumor cells for maintaining massive proliferation lies in the uncontrolled cell cycle. The fact that tumor cells lose cell-cycle checkpoints increases the chances of making mistakes during the replication of genetic material [84]. Previous studies showed that cisplatin, as the gold standard for tumor treatment, inhibits the cell cycle of tumor cells by arresting them in the G0/G1 or sub-G1 cell-cycle phase [85,86]. Experiments with dinuclear complexes of platinum(II) showed promising results, similar to cisplatin treatment, in blocking the cell cycle. Namely, Pt13 significantly increased the percentage of 4T1 cells arrested in the sub-G1 phase [59]. Analyses were also performed on the MDA-MB-468 human breast cancer cell line and, according to these, C2 not only arrested tumor cells in the sub-G1 phase, but also decreased the percentage of MDA-MB-468 cells in the Go/G1 phase [59]. Another study performed by Vasic et al. revealed that complexes such as Pt18–Pt20 block CT26 tumor cells in the G0/G1 phase. On the contrary, complexes Pt16 and Pt22 stop CT26 cells in the G2/M cell-cycle phase, in the same manner as the drug oxalplatin [57]. According to these results, different subtypes of dinuclear complexes of platinum(II) can act in different ways. Some of them are able to inhibit the tumor cell cycle by blocking the cells in the G0/G1 phase, and in this way progression into the S phase is prevented; the tumor cell cannot duplicate genetic material and thus proliferate, but this increases the chances of activating programmed cell death [87,88]. On the other hand, cells arrested in the G2/M phase may induce apoptosis of tumor cells, suggesting an even more efficient way of acting as a potential antitumor drug [89].

Delving deeper into the functioning of the cell cycle, it is important to know that cyclins and cyclin-dependent kinases, along with inhibitors of cyclin-dependent kinases (Cdk), play a major role in controlling the processes of cell proliferation and differentiation [90,91]. Nowadays, few drugs that act in a way to inhibit the cell cycle are used as a therapy against tumors [92]. A study by Zornic et al. revealed important information regarding the effect of dinuclear platinum(II) complexes (Pt12–Pt15, Figure 2) on cyclins. They showed that complex Pt13 significantly reduced expression of Cyclin D3 mRNA in 4T1 tumor cells. Moreover, the percentage of Cyclin E^+^4T1 cells was significantly decreased, compared to untreated cells [59]. Cyclin E, together with Cdk2, is crucial for the G1/S transition, which is a major step when the cells decide whether they enter into the S phase and start the process of DNA replication, or stay in the G1 phase. Abnormal activation of Cyclin E/CDK2 complex favors errors during DNA replication, thus promoting making of tumor cells [93]. Cyclin D, in the complex with Cdk4 and Cdk6, also plays a major role in the G1/S transition, and it is known that hyperexpression and abnormal activity of cyclin D induces uncontrolled cell proliferation, thus having an impact on tumor pathogenesis [94]. Besides affecting cyclins, the examined Pt13 dinuclear platinum(II) complex significantly increased expression of P27 mRNA in 4T1 cells, thus showing the same effect as cisplatin [59]. A key role of P27 is inhibition of the cyclin E-Cdk2 complex, and in that way, it regulates the G1 phase and prevents the cell from entering into the S phase [95]. Another molecule important for cell-cycle regulation is c-Myc. This transcriptional factor, a member of the Myc proto-oncogene family of proteins, is involved in major cell processes such as proliferation and differentiation, metabolism and cell death. C-Myc is able to stimulate cell proliferation and to inhibit antiproliferative molecules at the same time [96,97]. Many relevant studies confirmed continuous aberrant expression of c-Myc gene in more than 70 percent of human tumors, suggesting a very important role in tumorigenesis [97,98]. Zornic et al. investigated the effect of dinuclear platinum(II) complexes and revealed that Pt13 complex significantly decreased expression of c-Myc RNA in 4T1 cell in comparison to group of cells treated with cisplatin [59]. Moreover, they analyzed the effect of platinum complexes on the phosphatidylinositol-3 kinase (PI3K)–AKT pathway. pAKT coordinates proliferation, angiogenesis, and tumorigenesis, and thus serves as a significant target for antitumor pharmacy [99]. Although not as efficient as cisplatin, treatment with Pt13 significantly reduced the percentage of pAKT^+^ 4T1 cells in comparison to untreated cells [59].

Overall, dinuclear platinum(II) complexes remarkably reduced the possibility for tumor cells to proliferate by decreasing the expression of Ki67 and blocking the cell cycle in the Go/G1 and G2/M phase. Moreover, the suppression of cyclin D, cyclin E, c-Myc and AKT expression, together with increased activity of P27, makes these complexes attractive as an anticancer therapy.

## 6. Dinuclear Platinum(II) Complexes Inhibit Migratory Capacity of Target Tumor Cells

Alongside enhanced proliferation, unrestrained migration of a malignant cell is one of the hallmarks of a malignancy. To improve and boost cancer therapy, it is equally important to target both of these characteristics [100]. As the migration of a malignant cell causes various metabolic alterations, paving the way through the extracellular matrix for adaptations and proliferation in a tissue other than the one from which the malignant cell originates, it is, still, a great challenge to find a therapeutic agent that successfully impedes tumor cell migration effectively, at every step [101,102]. One of the well-studied tests to determine the inhibitory capacity of a therapeutic agent on tumor cell migration is the scratch assay [103]. The scratch assay is an in vitro assay where target cells are put to grow in a multiwall assay plate, after which a cell-free zone is created by scratching, after which the cell migration rate is monitored [104]. This simple assay is still one of the most effective ways to determine the migration potential of target cells [105].

As it is important to find new therapeutics to impede tumor cell proliferation, and it is, likewise, important to investigate therapeutic potential to impede cell migration, therefore potentially inhibiting tumor cell metastasis [106]. One of the most recently investigated newly synthesized anti-cancer agents, such as platinum-based therapeutics, is shown to be rather efficient in inhibiting tumor cell proliferation. When it comes to the migratory potential of malignant cells, platinum-based chemotherapeutics also exhibit beneficial effects. In a study by Bai et al., it has been shown that platinum-based anti-cancer chemotherapeutics significantly impede breast cancer cells’ migration, and therefore hinder breast cancer metastasis [106]. Our results align with these findings, suggesting that the administration of platinum-based chemotherapeutics has multiple effects on breast cancer expansion [107]. There is also evidence that platinum-based chemotherapeutic inhibits cancer cell migration in other tumor types, such as prostate cancer and oral cancer [108,109]. When it comes to prostate cancer, platinum-based compounds tend to downregulate the expression of epithelial–mesenchymal transition (EMT)-related gene expression, such as *BCL-2* and *BAX*, therefore decreasing the metastatic potential of cancer cells [108]. In oral carcinoma, cisplatin decreases the expression of E-cadherin in a dose-dependent manner [109].

As mentioned above, inhibition of tumor cell migration is one of the main mechanisms to suppress cancer progression, as it is crucial for metastasis. Platinum-based anti-cancer agents seem to express favorable effects, by utilizing various molecular pathways, putting them in the limelight for treatment of advanced malignant disease. However, more studies are required to further elucidate migration inhibition of platinum-based agents in order to combat more effectively the spread of a malignancy.

## 7. Dinuclear Platinum(II) Complexes Exhibit Strong Antitumor Effects with Reduced Side Effects In Vivo

Recent advances in cancer treatment have led to the development of dinuclear platinum(II) complexes, which are designed to target cancer cells more selectively, while minimizing side effects [110]. These complexes incorporate bridging ligands with aromatic rings containing nitrogen atoms, thus enhancing their ability to interact with cellular targets [59]. This section discusses the in vivo efficacy and safety profiles of two types of such complexes, highlighting their potential advantages over traditional chemotherapy agents like cisplatin.

In cancer research, considerable attention has been focused on dinuclear platinum(II) complexes that incorporate bridging ligands with aromatic rings containing two nitrogen atoms, specifically pyridazine and pyrazine (Figure 2). A particular complex with pyridazine, Pt14, demonstrated substantial cytotoxic effects against breast cancer cells in vitro [59]. However, in vivo studies on mice administered doses of 3 milligrams per kilogram and 6 milligrams per kilogram revealed that this complex unexpectedly enhanced tumor growth, compared to cisplatin. Despite these results, the lower dose resulted in improved survival rates from the 18th day onward, while the higher dose saw a decline in survival rates, highlighting a complex interaction between tumor growth and survival outcomes [59]. In contrast, dinuclear platinum(II) complexes featuring pyrazine ligands (Pt16–Pt23) significantly reduced tumor volumes in a heterotopic mouse model of colon cancer, similar to the effects observed with oxaliplatin, a standard component in colorectal cancer treatment protocols such as FOLFOX (Folinic Acid, Fluorouracil, and Oxaliplatin) [57,111]. At necropsy, the primary tumors in mice treated with these pyrazine complexes or oxaliplatin weighed significantly less, compared to untreated controls. Additionally, there was a significant reduction in metastatic foci in the livers and lungs. Only 10% of treated mice developed lung metastases, which were fewer and smaller in size, compared to 50% of the untreated group. Liver metastases were also notably less frequent in treated groups [57]. Furthermore, the complexes were well tolerated, with no significant differences in the levels of Alanine Aminotransferase (a marker of hepatotoxicity), urea, and creatinine (markers of nephrotoxicity) between the treated and untreated groups. These findings underscore the complexes’ efficacy in targeting cancer cells while preserving liver and kidney health [57].

## 8. Antimicrobial Activity of Dinuclear Platinum(II) Complexes

The relationship between infectious agents and the host in oncology is a complex interaction that has not been studied enough. The significant spread of resistant strains and the formation of bacterial biofilms is a serious health concern [112,113]. It is increasingly clear that producing effective antibiotics is not enough to treat infectious diseases. This highlights the need for ongoing testing of anticancer and antimicrobial activity of other compounds, such as the platinum complex, for use in clinical practice.

Cisplatin was initially discovered as a substance that inhibits the division of Escherichia coli bacteria [114]. Clinical trials have demonstrated that platinum complexes have strong antimicrobial properties [115]. A recent systematic study examined 906 metal complexes and found that they have a 10-fold higher hit rate against critical ESCAPE (*Enterococcus faecium*, *Staphylococcus aureus*, *Klebsiella pneumoniae*, *Acinetobacter baumannii*, *Pseudomonas aeruginosa*, and *Enterobacter* spp.) bacterial pathogens and fungi compared to organic molecules. Among 63 platinum-containing compounds, 43% showed antimicrobial potential. Further evaluation identified 18 platinum compounds with antimicrobial activity, without toxic effects on mammalian cells at the same concentration [116]. Frei et al. [116] investigated the antibacterial properties of platinum cyclooctadiene (COD) complexes and found that Pt(COD)Cl2 (Pt1) and Pt(COD)I2 (Pt2) complexes exhibit excellent antibacterial activity against a wide panel of Gram-positive strains, particularly *S. aureus*, including methicillin-resistant *Staphylococcus aureus* and vancomycin-resistant strains, as well as *S. epidermidis* and *B. subtilis*. It is worth noting that, despite their similarity to cisplatin, these compounds did not show any cytotoxicity towards human cells, even at the highest concentrations tested [117]. Jawad et al. [118] demonstrated that the platinum(IV) complex with a bidentate ligand of 4-amino-5-(3,4,5-trimethoxyphenyl)-4I-1,2,4-triazole-3-thiol showed high antimicrobial activity against bacteria *Staphylococcus aureus* (Gram-positive) and *Escherichia coli* (Gram-negative).

## 9. Conclusions

**Figure 5:**
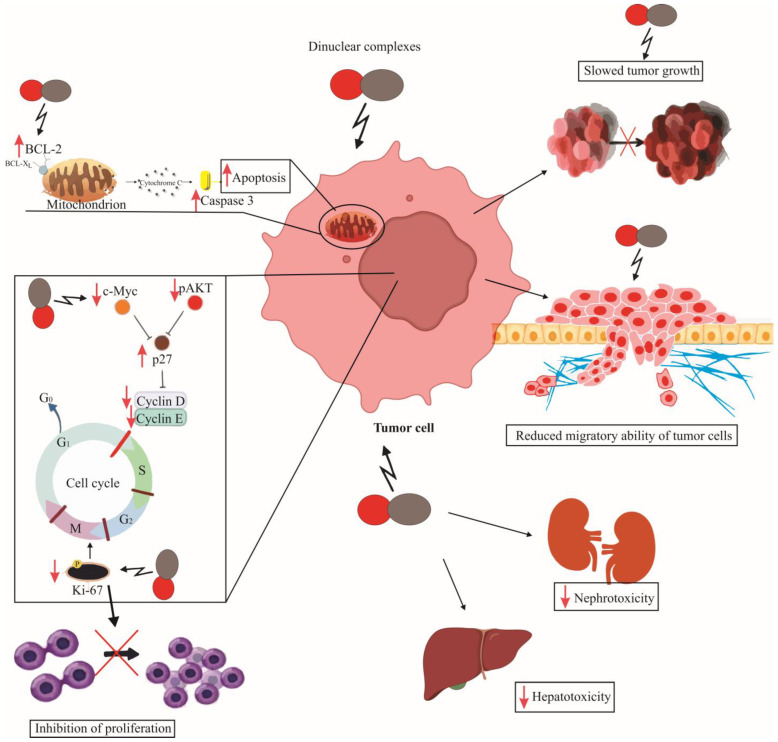
Effects of Dinuclear platinum(II) Complexes on Tumor Cells. The figure depicts how dinuclear complexes reduce the expression of the anti-apoptotic BCL-2 molecule on mitochondria, consequently increasing the expression of Caspase 3 in tumor cells. They enhance the expression of the cyclin-dependent kinase inhibitor p27, while decreasing the expression of c-Myc and pAKT; this is accompanied by a reduction in the expression of cyclins D and E, thereby arresting the cell cycle in the G0/G1 phase. The expression of Ki67 is reduced, further indicating the inhibition of proliferation. Consequently, the migratory ability of tumor cells is decreased, leading to slower primary-tumor growth, with reduced hepatotoxicity and nephrotoxicity observed.

This comprehensive review elucidates the significant strides made in the development of dinuclear platinum(II) complexes as potential anticancer agents. The insights from various studies underscore the promising cytotoxic activities of these complexes against a range of cancer cell lines. Unlike traditional platinum-based therapies, which often present severe side effects and drug resistance, dinuclear complexes offer a distinct mechanism of action. This mechanism includes unique DNA-binding patterns that contribute to their effectiveness while potentially reducing adverse effects. Furthermore, these complexes demonstrate enhanced selectivity and potency, indicative of their capability to induce apoptosis and inhibit cell proliferation in a targeted manner (Figure 5). However, despite their profound in vitro efficacy, the translation of these complexes into clinical use necessitates rigorous in vivo testing to ascertain their therapeutic indices and to optimize their pharmacological profiles. As research progresses, dinuclear platinum(II) complexes continue to represent a compelling avenue for novel anticancer drug development, aligning with ongoing efforts to refine chemotherapy and improve patient outcomes.

## Figures and Tables

**Table 1:** Platinum(II) complexes approved as anticancer drugs for human use.

General Name	Chemical Structure	Trade Name	Year of Approval
cisplatin	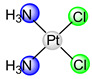	platinol	1978
carboplatin	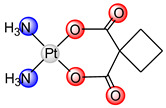	paraplatin	1989
oxaliplatin	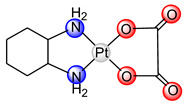	eloxatin	2002
nedaplatin	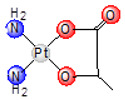	aqupla	1995
heptaplatin	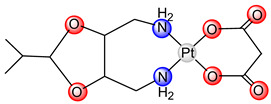	SunPla	1999
laboplatin	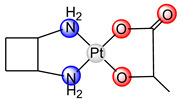	-	2010

## Data Availability

This review article integrates findings from previously published research and does not introduce original empirical data. Complete citations of all referenced sources are included within the text. Readers and researchers seeking to access the original studies and datasets may refer to the cited publications or contact the authors of those works directly. For further information or specific questions, please contact the corresponding authors of this review.
